# Results of 1470 nanometre diode laser assisted stapedotomy

**DOI:** 10.1017/S002221512510279X

**Published:** 2025-11

**Authors:** Ulrica Thunberg

**Affiliations:** Department of Otorhinolaryngology, Örebro University Hospital, Sweden and Faculty of Medicine and Health, Örebro University, Örebro, Sweden

**Keywords:** Stapedotomy, diode laser, laser, otosclerosis, surgery

## Abstract

**Objectives:**

Few studies have investigated stapedotomy using 1470 nm diode laser, and the present article contributes with clinical experience.

**Methods:**

A retrospective analysis was conducted to investigate hearing performance of 22 patients undergoing 1470 nm diode laser-assisted primary stapedotomy.

**Results:**

In 8/22 cases, accidental breaches to the inner ear by the laser and in 14/22 cases the stapedotomy was performed as planned only by drill. Air–bone gap and loss of sensorineural hearing were low and with no significant differences for groups at follow-up. No patients with breaches by laser reported new or worse tinnitus.

**Conclusion:**

Accidental breaches in the stapes footplate using this diode laser did not appear to equate with inner ear damage within this limited cohort. Hearing outcomes were not significantly affected. These findings should be interpreted with caution. Further studies evaluating this laser wavelength in stapedotomy is required.

## Introduction

Otosclerosis is a common cause of conductive hearing loss.^1^ The disease is progressive, and characterised by ossification of the stapes footplate that hinders sound waves from reaching the inner ear. If the disease solely affects the footplate, a mechanical hearing impairment occurs; but as time passes the cochlea will often be involved, causing additional sensorineural hearing loss.^2^ Stapedotomy, which was first introduced by Rosen in 1953, is the main surgical method which aims to restore the movement of the ossicular chain. During stapedotomy, the stapes superstructures are removed, and a perforation is created in the stiff footplate where a prosthesis is placed to transmit sound waves to the inner ear.

Creating an opening to the inner ear is a delicate procedure that involves a risk of sensorineural hearing loss, although most cases have excellent hearing results with few complications. Different techniques and use of micro instruments have been suggested to minimise the risk of injury to the inner ear. In recent years, a variety of lasers have been used for stapedotomy, including argon, CO_2_, potassium-titanyl-phosphate (KTP) and diode lasers of different wavelengths.^3^ The goal is to be as gentle as possible to preserve the inner ear. Laser has the advantage of being a non-touch technique that offers a high precision for incision, but its effects on the inner ear are not fully understood. The laser might have thermal and acoustic effects on the inner ear^4^; but on the other hand, drilling might cause noise and mechanical trauma to the inner ear.^2^^,^^5^^,^^6^ Comparative laser studies have not shown a clear difference in hearing results.^7^^–^^9^

Since 2018, a 1470 nm diode laser has been used in combination with a microdrill in stapedotomy procedures at Örebro University Hospital, Sweden. As accidental fenestration occasionally occurred while thinning the footplate with laser. Few studies have presented results using this laser in stapedotomy, and the present article provides valuable clinical insights.

The aim of the current study was to evaluate the outcome of small breaches when using a 1470 nm diode laser in stapedotomy.

## Materials and methods

### Patients

This was a retrospective study based on data from medical files covering all stapedotomies performed at Örebro University Hospital during 2018–2021. No stapedectomies were performed during this period. Patient cases were identified by a specific surgical code for stapedotomy, and all identified patients were sent a letter inviting them to participate along with information about the study and a request for their signed consent.

Information was collected about age, gender, operated ear, date of surgery, type and length of prosthesis connecting to the inner ear, laser effect on the stapes plate and fenestration and pre-operative and post-operative (from late follow-up: one year) audiology data. Post-operative tinnitus, vertigo and taste disturbance were noted as well as any peri-operative or post-operative adverse event. Cases were pseudonymised and given a unique study identification (ID) number. Information about pre-operative examination by otomicroscopy and pure tone audiometry was available for all patients.

### Pre-operative and post-operative data

Pure tone audiometry was performed pre-operatively and at the one-year follow-up. As routine, the operated ear was tested at frequencies of 500, 1000, 2000, 3000, 4000, 6000 and 8000 Hz for air conduction (AC) and at 500, 1000, 2000, 3000 and 4000 Hz for bone conduction (BC). Pure tone average (PTA^4^) for AC and BC as well as the air–bone gap (ABG) was defined as the mean value for 500, 1000, 2000 and 3000 Hz. High frequency hearing was defined as mean value 4000, 6000 and 8000 Hz.

### Surgery

All stapedotomies were performed by a single surgeon under general anaesthesia. Using an endaural approach, a tympanomeatal flap was raised and the annulus was elevated, the chorda tympani preserved. Stapedial fixation was confirmed by delicate palpation of the ossicular chain. The scutum was drilled or curetted. The incudostapedial joint was divided by an angled needle, and the stapedial tendon was cut by scissor or divided with a 1,470 nm diode laser attached to a handpiece. The stapedial superstructure was removed using a laser fibre (2–3 W) applied to the posterior crus and when accessible also to the anterior crus. What was left of the anterior crus was fractured close to the stapes footplate, and the loose superstructure was removed with an angled needle. With effect 1 or 2 W, the footplate was thinned and then either penetrated with a 0.6 mm Skeeter diamond drill (pD group) or because of accidental breaches by the laser; in the latter case, the penetration was completed by drill (pLD group). The distance between the stapes footplate and the lateral edge of the long process of the incus was measured with a measuring rod, a Fisch-type platinum/fluoroplastic piston prosthesis (Olympus) or titanium K-Piston (Heinz Kurtz) was placed into the perforation and the loop of the prosthesis was manually crimped around the long process of the incus. The stapedotomy procedure was completed in all cases.

### Statistical analysis

Descriptive statistics included the calculation of mean and median values, ranges and standard deviations (SDs). The Wilcoxon rank sum test was used for statistical analysis of continuous data, and Fisher’s test was used for non-parametric data. A *p*-value of less than 0.05 was considered statistically significant.

## Results

### Patient characteristics

Despite the pandemic situation, 24 patients could be treated with stapedotomy for otosclerosis during 2018–2021. One patient with a cognitive impairment was excluded from this study due to inability to provide their signed informed consent, and one did not agree to participate, leaving 22 stapedotomies for inclusion. Of these, 14 were performed solely by drill, while in the remaining eight cases, the laser accidentally perforated the stapes footplate and the stapedotomy was then completed by drill. All stapedotomies were performed under general anaesthesia as a daycare procedure. None were revision surgery. There was a mean of 508 days between surgery and post-operative audiometry, and a mean of 88 days between pre-operative audiogram and surgery. A Fisch-type piston was used in 20 cases and a K-Piston in the remaining two. A piston width of 0.4 mm was used in all cases. General data are presented in Table 1.

**Table 1.** Descriptive variables for patients undergoing primary stapedotomy

Parameter	pD	pLD	Total
	*n* = 14 (%)	*n* = 8 (%)	*n* = 22 (%)
Age (year) mean ± SD	51±18	47±11	49±16
Gender			
Female	9 (64)	5 (63)	14 (64)
Male	5 (36)	3 (37)	8 (36)
Operated ear			
Surgery left	5 (36)	3 (37)	8 (36)
Surgery right	9 (64)	5 (63)	14 (64)
Wattage at footplate			
≥2.0 W	12 (86)	7 (88)	19 (86)
<2.0 W	2 (14)	1 (12)	3 (14)
Length of prosthesis (mm)			
4.50	8 (57)	5 (63)	13 (59)
4.75	4 (29)	2 (25)	6 (27)
5.0	2 (14)	1 (12)	3 (14)
Tinnitus at follow-up*			
No tinnitus	9 (64)	2 (25)	11 (50)
Unchanged tinnitus	3(21)	5 (63)	8 (36)
Less tinnitus	1 (7)	1 (12)	2 (9)
Worse tinnitus	1 (7)	0 (0)	1 (5)
Taste disturbance at follow-up*			
Yes	2 (14)	0 (0)	2 (9)
No	11 (79)	8 (100)	19 (86)
Post-operative vertigo			
Yes	0 (0)	1 (10)	1 (5)
No	14 (100)	7 (90)	21 (95)

Abbreviations: pD, penetration of stapes footplate by drill; pLD, accidental penetration of stapes footplate by laser which was then completed by drill.

* Follow-up was conducted approximately one year after surgery.

### Pre-operative evaluation

In the total group, pre-operative mean AC-PTA^4^ was 66 dB, hearing Level (HL) (SD: 17), mean BC-PTA^4^ was 33 dB, HL (SD: 17), and mean ABG-PTA^4^ was 33 dB, HL (SD: 11). Pre-operative hearing level for AC-PTA^4^ was better in the pLD group than in the pD group (Table 2).

**Table 2.** Pre-operative and post-operative audiometry data for the operated ear

Parameter	pD	pLD	Total	*p*-Value*
	*n* = 14	*n* = 8	*n* = 22	
Pre-operative data, db HL				
AC-PTA^4^				
Mean ± SD	70 ± 17	60 ± 15	66 ± 17	0.219
Median (range)	68 (61)	58 (45)	64 (61)	
BC-PTA^4^				
Mean ± SD	36 ± 20	28 ± 8	33 ± 17	0.339
Median (range)	38 (56)	28 (26)	29 (56)	
ABG PTA^4^				
Mean ±SD	33 ± 12	32 ± 10	33 ± 11	0.811
Median (range)	31 (35)	29 (25)	29 (35)	
Post-operative data, dB HL				
AC-PTA^4^				
Mean ±SD	39 ± 22	30 ± 16	36 ± 20	0.432
Median (range)	32 (64)	25 (50)	29 (64)	
BC-PTA^4^				
Mean ±SD	31 ± 21	23 ± 12	29 ± 18	0.608
Median (range)	24 (61)	21 (39)	23 (61)	
ABG PTA^4^				
Mean ±SD	8 ± 7	7 ± 4	7 ± 6	0.945
Median (range)	5 (25)	6 (14)	5 (25)	

Abbreviations: ABG, air–bone gap; AC, air conduction; BC, bone conduction; dB HL, decibel hearing level; pD, penetration of stapes footplate by drill; pLD, accidental penetration of stapes footplate by laser which was then completed by drill; PTA^4^, four-frequency pure tone average (500, 1000, 2000 and 3000 Hz).

* Wilcoxon rank-sum test.

### Post-operative evaluation

There were no statistically significant differences between the pLD and pD groups in the magnitude of change in AC, BC or ABG, nor in the overall hearing results in terms of successful surgery (Table 3).

### Air conduction

**Figure 1.**
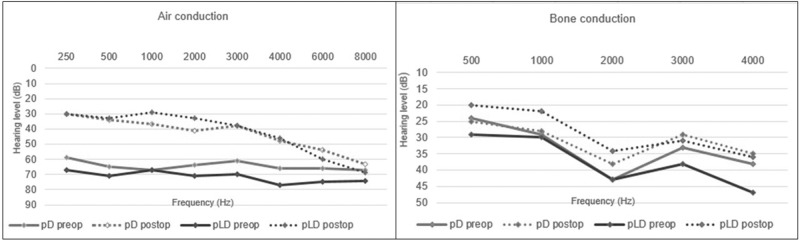
Pre-operative and post-operative air conduction (250–8000 Hz) and bone conduction (500–4000 Hz) hearing levels in the operated ear. pD, penetration of stapes footplate by drill (*n* = 14); pLD, accidental penetration of stapes footplate by laser which was then completed by drill (*n* = 8). Missing values at 6000 Hz and 8000 Hz for one pD and one pLD. Lines are drawn between marks for the hearing level achieved at each frequency.

At follow-up after surgery, the mean AC-PTA^4^ for all patients was 36 dB, HL (SD: 20). There was no statistically significant difference in mean post-operative AC-PTA^4^ between pLD patients and pD patients. An AC-PTA^4^ hearing gain of greater than or equal to 20 dB was achieved in 11/14 (79 per cent) of the pD group and 7/8 (88 per cent) of the pLD group (Table 3). One pLD patient showed a large ABG and unchanged AC post-operatively, along with prosthesis dysfunction. This patient declined revision surgery at that time. High-frequency AC hearing level after surgery was with no significant change post-operative in both groups (Figure 1).

**Table 3.** Overall hearing results

Parameter	pD (*n* = 14) n (%)	pLD (*n* = 8) n (%)	*p*-Value
AC-PTA^4^ gain ≥ 20 dB	11 (79)	8 (100)	0.236
BC-PTA^4^ loss ≤ 5 dB	13 (93)	7 (88)	0.606
ABG-PTA^4^ closure to ≤ 10 dB	11 (79)	7 (88)	0.535

Abbreviations: ABG, air–bone gap; AC, air conduction; BC, bone conduction; pD, penetration of stapes footplate by drill; pLD, accidental penetration of stapes footplate by laser which was then completed by drill; PTA^4^, four-frequency pure tone average (500, 1000, 2000 and 3000 Hz).

### Bone conduction

Average BC-PTA^4^ at follow-up was 29 dB, HL (SD: 18), with no statistically significant difference between the pLD and pD groups. A hearing loss of less than 5 dB after surgery was seen in 93 per cent of the pD group and 88 per cent of the pLD group; this difference was not statistically significant.

#### Air–bone gap

Average ABG at follow-up was 7 dB (SD 6). A closure to less than or equal to 10 dB was obtained in 82 per cent of all operated ears (Table 3), and in 86 per cent of cases after exclusion of the patient with a non-functioning prosthesis. There was no statistically significant difference in post-operative ABG gain between the pLD and pD groups.

### Unexpected events

Unexpected events occurred in three cases. One patient was found to have a slight stiffness of the incus, whereupon the ossicular chain was mobilised and the stapedotomy was performed only by drill. This patient had a post-operative gain of 43 dB AC-PTA^4^ and 4 dB ABG-PTA^4^ at follow-up after one year. One pLD patient suddenly experienced acute vertigo one week after surgery during physical effort. He recovered fully in a few days; at follow-up, his hearing gain was 32 dB AC-PTA^4^ and 4 dB ABG-PTA^4^, and the inner ear was spared. In another patient, the measuring stick accidently penetrated a thin stapes footplate, whereupon perforation was completed by drill. At follow-up, this patient had a post-operative AC-PTA^4^ gain of 17 dB (to 38 dB) and an ABG of 26 dB PTA^4^. Very few patients reported taste disturbances.

## Discussion

Stapedotomy is the standard surgical intervention for the treatment of otosclerosis worldwide. Its purpose is to improve hearing while causing as little harm as possible to the inner ear during the surgical procedure. Many studies have been published regarding the use of different lasers in stapes surgery, though few have investigated diode lasers with wavelengths of 808, 812 and 980 nm, and even fewer have investigated the 1470 nm diode laser.

Koenraads *et al*. constructed an experimental model using fresh frozen human temporal bone to study the effects of a 1470 nm laser, and found high absorption of the wavelength in water, thermal effects, pressure waves and formation of vaporisation bubbles above the stapes footplate. Acoustic effects were limited.^4^ Caution is to be exercised when using this laser in stapes surgery and an occasional breach is an adverse event. In our cohort of patients, an accidental breach in the footplate induced by this laser did not cause an inner ear catastrophe, but it is important to be aware that this cannot be regarded with equal effect as a complete penetration by laser. A systematic review and meta-analysis by Bartel *et al*. included 1531 patients undergoing stapes surgery, 978 of whom were operated with drilling technology and 553 with laser (CO_2_ laser or KTP laser). No significant differences were found in the achieved hearing results between the groups.^10^ A study by Pauli *et al*. based on the Swedish national quality register for otosclerosis suggested that a combination of laser (CO_2_ or KTP) and microdrill was favourable for hearing results, but diode laser was not specified as a separate group.^7^ Navarrette *et al*. reported their experience with a 980 nm diode laser in six patients and found no post-operative decrease in bone conduction and a spared inner ear.^11^ Parida *et al*. presented a study of 60 stapedotomy patients randomly assigned to either 980 nm diode laser or manual penetration with a Shea pick.^12^ There was no difference in hearing outcomes, and no patient ended up with sensorineural hearing loss. In another study using continuous mode at 1.5 W when perforating the footplate, there were cases with sensorineural hearing loss.^13^

In a randomised clinical trial, the surgical effects of the conventional microdrill technique and the 980 nm diode laser were compared. Patients with otosclerosis were randomly assigned to either the microdrill group (*n* = 69) or the 980 nm diode laser group (*n* = 62). Post-operative hearing improvement for the two groups was equivalent, with no significant differences in complications.^14^ Surgeons have been reserved to use the 1470 nm laser, and published clinical experience is lacking. In the present study, we retrospectively investigated the results of accidental laser breaches of the stapes footplate when using a 1470 nm diode laser during stapedotomy. We found that the hearing results were equivalent for patients in whom a small breach occurred in the footplate compared to those with penetration solely by use of a microdrill. At follow-up, the loss of sensorineural hearing was low in both groups, and no significant differences were seen between the groups. Overall, 91 per cent had a bone conduction loss of less than 5 dB and a mean ABG of 7 dB at follow-up after approximately one year. Eleven of 22 patients reported no tinnitus at follow-up, and no patients with laser penetration into the inner ear reported new or worse tinnitus.

The present study is limited due to its small sample size; moreover, the wattage used at the footplate varied between patients, which might have affected the results. However, the consecutive inclusion of patients is a strength. The findings in this retrospective study contribute with novel experiences regarding the clinical audiological effect of breaches of the stapes footplate by diode laser 1470 nm.

- Few studies have investigated stapedotomy using 1470 nm diode laser, and the present article contributes relevant clinical experience.
- A consecutively included cohort of 22 patients undergoing 1470 nm diode laser assisted stapedotomy was retrospectively studied.
- Small accidental breaches of the stapes footplate with the laser did not affect hearing results in comparison to results after perforation only by microdrill.

- No patients with penetration of laser reported new or worse tinnitus.

- These findings should be interpreted with caution. Further studies evaluating this laser wavelength in stapedotomy is required.

## Conclusion

In this retrospective study of a consecutively included small cohort of primary stapedotomy patients, hearing results were not statistical different when comparing cases with accidental breaches of the stapes footplate by a 1470 nm diode laser and conventional stapedotomy by drill. The inner ear was not harmed by the breaches by the laser. This study needs cautious interpretation of the results, and further investigation is required to gain reliable knowledge regarding this wavelength of laser and how it affects the inner ear.
